# Supplementary material for: Trabeculae microstructure parameters serve as effective predictors for marginal bone loss of dental implant in the mandible
Source: Sci Rep. 2020 Oct 28;10:18437. doi: 10.1038/s41598-020-75563-y (PMC7595041; doi:10.1038/s41598-020-75563-y)
Supplement: Supplementary file 1 — Supplementary Legends. [file 41598_2020_75563_MOESM1_ESM.docx]

Figure S1. Reference lines for measurement of marginal bone loss around dental implants. R = reference line; X = implant diameter; L = implant length; M = mesial measurement; D = distal measurement.

Figure S2. Measurement of peri-implant bone morphological parameters. (a) Radiograph grayscale was selected by the principle of showing trabecular bone (marked in red) completely. (b) Five sequential ROI layers adjoining the implant were selected as VOI of the peri-implant alveolar bone (marked in red). Five sequential ROI layers away from the implant were chosen as VOI of the normal adjacent alveolar bone (marked in blue).
