# Supplementary figures and images for: Trabeculae microstructure parameters serve as effective predictors for marginal bone loss of dental implant in the mandible
Source: Sci Rep. 2020 Oct 28;10:18437. doi: 10.1038/s41598-020-75563-y (PMC7595041; doi:10.1038/s41598-020-75563-y)

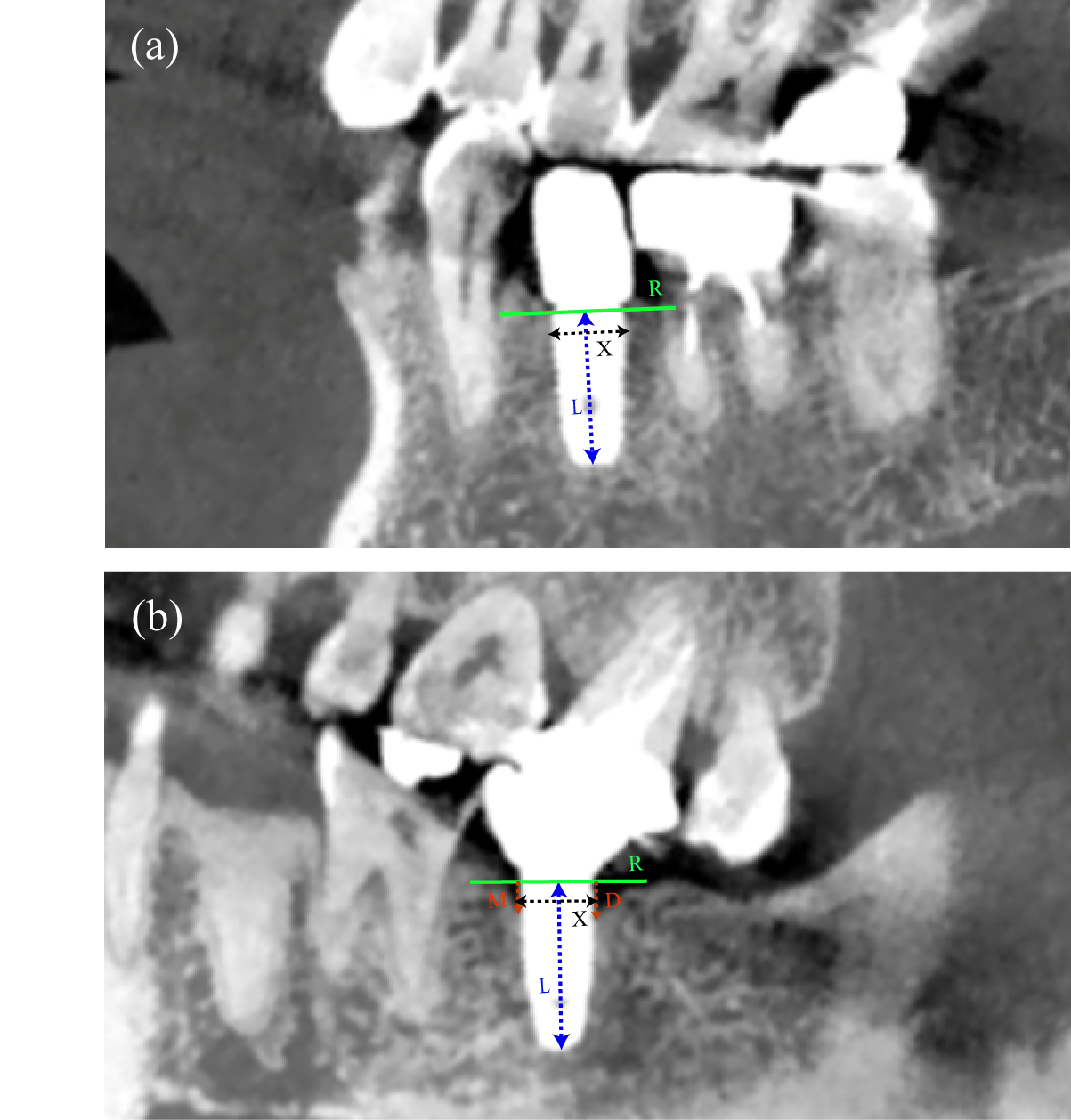

Supplement: Supplementary file 2 — Supplementary Figure S1. [file 41598_2020_75563_MOESM2_ESM.tif]

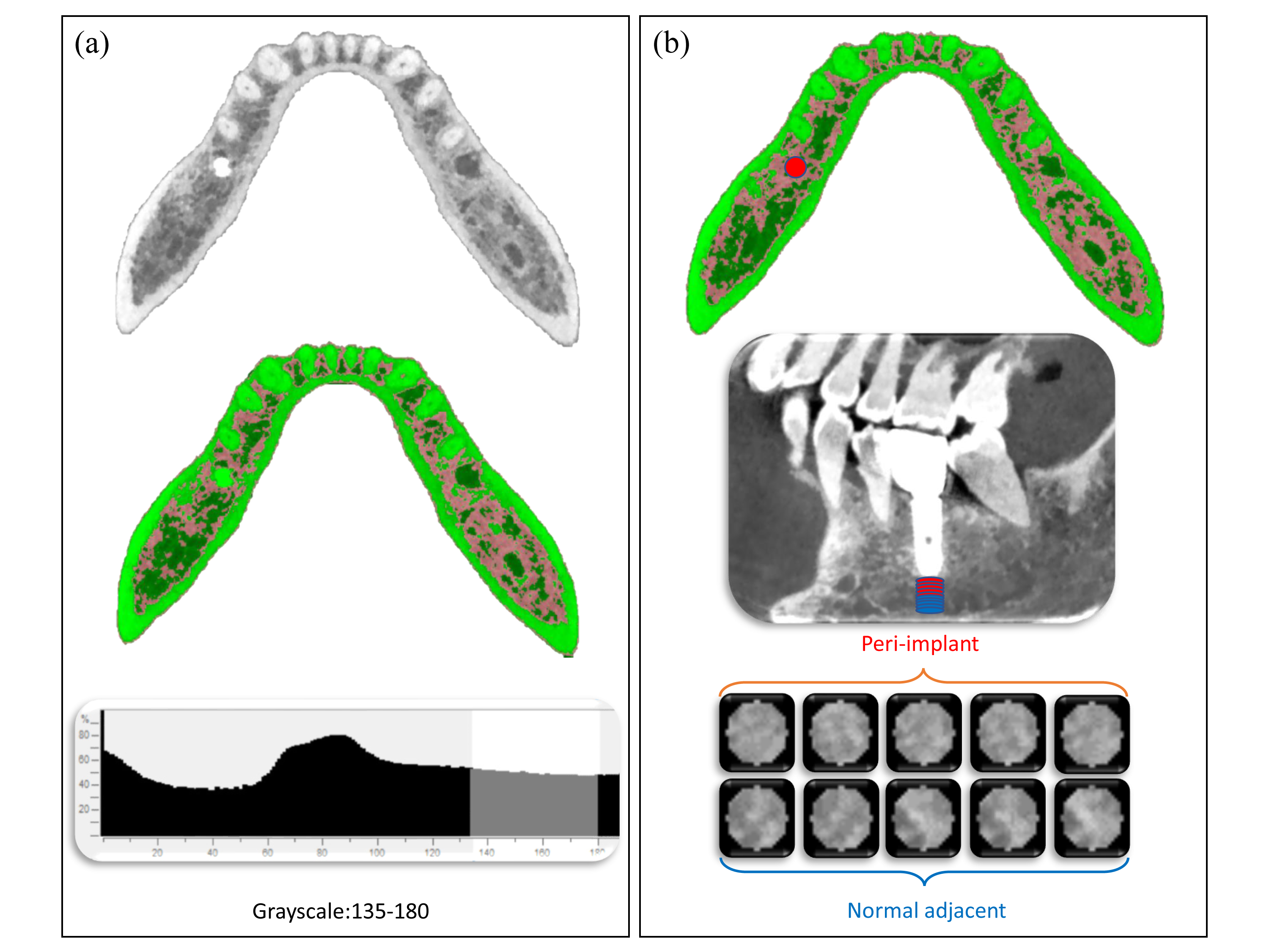

Supplement: Supplementary file 3 — Supplementary Figure S2. [file 41598_2020_75563_MOESM3_ESM.tif]
